# Neuroimaging Findings in a Patient with Anti-IgLON5 Disease: Cerebrospinal Fluid Dynamics Abnormalities

**DOI:** 10.3390/diagnostics12040849

**Published:** 2022-03-30

**Authors:** Daniele Urso, Roberto De Blasi, Antonio Anastasia, Valentina Gnoni, Valentina Rizzo, Salvatore Nigro, Benedetta Tafuri, Carlo Maria Iacolucci, Chiara Zecca, Maria Teresa Dell’Abate, Francesca Andreetta, Giancarlo Logroscino

**Affiliations:** 1 Center for Neurodegenerative Diseases and the Aging Brain, Department of Clinical Research in Neurology, University of Bari ‘Aldo Moro’, “Pia Fondazione Cardinale G. Panico”, 73039 Tricase, Italy; gnoni.vale@gmail.com (V.G.); salvatoreangelo.nigro@gmail.com (S.N.); benedetta.tafuri@gmail.com (B.T.); dott.iacolucci@gmail.com (C.M.I.); chiarazecca.cz@gmail.com (C.Z.); dellabatemariateresa@gmail.com (M.T.D.); 2 Department of Neurosciences, Institute of Psychiatry, Psychology and Neuroscience, King’s College London, De Crespigny Park, London SE5 8AF, UK; 3 Department of Diagnostic Imaging, Pia Fondazione di Culto e Religione “Card. G. Panico”, 73039 Tricase, Italy; robertodeb55@gmail.com (R.D.B.); antonio.anastasia@inwind.it (A.A.); vale.rizzo86@gmail.com (V.R.); 4 Department of Basic Medicine, Neuroscience, and Sense Organs, University of Bari ‘Aldo Moro’, 70124 Bari, Italy; 5 Neurology IV-Neuroimmunology and Neuromuscular Disease Unit, Fondazione I.R.C.C.S. Istituto Neurologico Carlo Besta, 20133 Milan, Italy; francesca.andreetta@istituto-besta.it

**Keywords:** anti-IgLON5 disease, neuroimaging, PSP, MRI, nuclear medicine, cerebrospinal fluid dynamics

## Abstract

Anti-IgLON5 disease is a recently described autoimmune neurodegenerative disorder characterized by insidious onset, slow progression and a variety of neurological features. Neuroimaging in most patients with anti-IgLON5 disease is normal or shows nonspecific findings. Here, we report a case of anti-IgLON5 disease presenting with parkinsonism, falls, sleep problems with severe nocturnal dyspnea attacks, dysphagia, and dysautonomia. Imaging findings were initially suggestive of progressive supranuclear palsy. An altered cerebrospinal fluid dynamic was found on an MRI as well as high-convexity hyperperfusion on a brain SPECT. Further case descriptions with neuroimaging are required to characterize cerebral and cerebrospinal fluid dynamics abnormalities in this rare condition.

A 63-year-old man, with no previous history of neurological disorder, presented with a one-year history of slowness of movement as well as gait difficulties with postural instability and complained of episodes of falls and swallowing problems. He also reported a five-year history of vivid dreams with dream-enacting behaviour, suggestive of REM sleep behaviour disorder (RBD), and a two-year history of erectile dysfunction. Six months before the visit, he developed laryngeal stridor during sleep and severe nocturnal dyspnea attacks resulting in acute respiratory failure requiring intubation and subsequent tracheostomy. His family history was negative for neurodegenerative disorders. A neurologic examination showed horizontal gaze-evoked nystagmus, bradykinesia, rigidity, and mild rest and postural tremor that was more evident on the left side. He had reduced right arm swing when walking and a shuffling gait. Neuroimaging studies were carried out to differentiate atypical parkinsonian syndromes and to rule out alternative diagnoses. A structural brain 3T MRI showed temporal atrophy with relative preservation of other cortical areas and demonstrated high-convexity tight sulci (Figure 1). Subcortically, midbrain atrophy was evident (the “hummingbird” sign). An arterial spin labelling (ASL)-MRI documented relative hyperperfusion of the high-convexity area with relative preservation of other brain regions. Brain perfusion SPECT imaging confirmed relative hyperperfusion in the high-convexity area. [123I] FP-CIT SPECT demonstrated a bilateral dopaminergic nigrostriatal denervation more prominent in the right putamen. The magnetic resonance parkinsonism index (MRPI 2.0) [1], a reliable imaging morphometric marker for the diagnosis of progressive supranuclear palsy (PSP), was manually calculated and was suggestive of PSP. A routine CSF analysis was unremarkable, and CSF amyloid beta (Aβ42) and tau (total and phosphorylated tau) levels were normal. Although the clinical and radiological presentation was suggestive of PSP, some elements were atypical. First, vertical supranuclear palsy or slow velocity of the vertical saccades, which are clinical features with high diagnostic relevance for PSP, were absent in this patient. Second, laryngeal stridor is rare in PSP and is typically present in other atypical parkinsonisms, such as multiple system atrophy. Recently, a new disorder characterized by nonREM and REM parasomnias, stridor, and gait instability resembling PSP (PSP-like syndrome) has been described, which occurs in association with antibodies against extracellular epitopes of IgLON5, a neuronal cell adhesion protein. Autoantibodies for IgLON5 IgG were tested in both the serum and CSF using a commercially available cell-based assay and returned positive in our patient (Supplementary Figure S1).

Anti-IgLON5 disease is a very rare clinical entity characterized by distinctive sleep disorders associated with a broad variety of neurological symptoms, such as parkinsonism with gait instability, bulbar symptoms, and dysautonomia. This condition has been first described in 2014 [2], and since then, more than 60 cases of anti-IgLON5 disease have been reported in the literature [3]. Its pathophysiology still remains unknown, but it seems to be the result of a combination of both autoimmune and neurodegenerative processes. Characteristic neuropathologic findings include a lack of inflammatory infiltrates, neuronal loss, gliosis, and neuronal accumulation of hyperphosphorylated tau protein (both 3-repeat and 4-repeat isoforms) found predominantly in the hypothalamus and the tegmental brainstem nuclei [2]. MRI findings are normal or nonspecific in 81.8% of cases [3,4,5]. The most frequent abnormality is brainstem atrophy [6]. Ioflupane SPECT abnormalities, though scarcely described, have been reported in anti-IgLON5 disease, probably reflecting nigrostriatal dopaminergic degeneration in the context of the tauopathy component of the disease [7]. In this case, midbrain atrophy documented by a structural MRI and dopaminergic denervation shown by a dopaminergic SPECT oriented initially for a diagnosis of PSP. Furthermore, the imaging morphometric marker MRPI was also indicative of PSP. Interestingly, the MRI also demonstrated “high-convexity tight sulci”, an established neuroimaging biomarker of CSF dynamics disorders and defined as compression of sulci at the vertex, enlarged CSF spaces in the Sylvian fissure, and ventriculomegaly. Although “high-convexity tight sulci” was originally identified in individuals with symptomatic normal-pressure hydrocephalus (NPH), it has been also found in other conditions [8]. Similarly, hyperperfusion of the high-convexity area on perfusion imaging has been previously linked to NPH [9]. We believe that relative convexity hyperperfusion in our case is apparent and may reflect the increased gray matter density of the convexity, as it has been demonstrated in NPH [9]. Although CSF dynamics abnormalities have been observed in other conditions [8], to the best of our knowledge, this is the first time that neuroimaging reveals CSF dynamics problems in anti-IgLON5 disease. This case highlights the fact that anti-IgLON5 disease can exhibit the clinical and radiological changes seen in PSP patients and that neuroimaging markers of CSF dynamics problems (“high-convexity tight sulci” on an MRI and hyperperfusion of the high-convexity on both an ASL-MRI and a perfusion SPECT) may be found in IgLON5. Further case descriptions and case−control neuroimaging studies are warranted to characterize neuroimaging abnormalities in this condition. Neuroimaging studies are also required because CSF dynamics abnormalities are detectable in vivo only, and neuropathological studies cannot be used to investigate this phenomenon. Although clinical presentation may be very distinctive, imaging biomarkers may potentially assist the diagnosis of this rare condition. The early recognition of anti-IgLON5 diseases is essential as immunotherapy seems to be crucial for clinical outcomes.

## Figures and Tables

**Figure 1:**
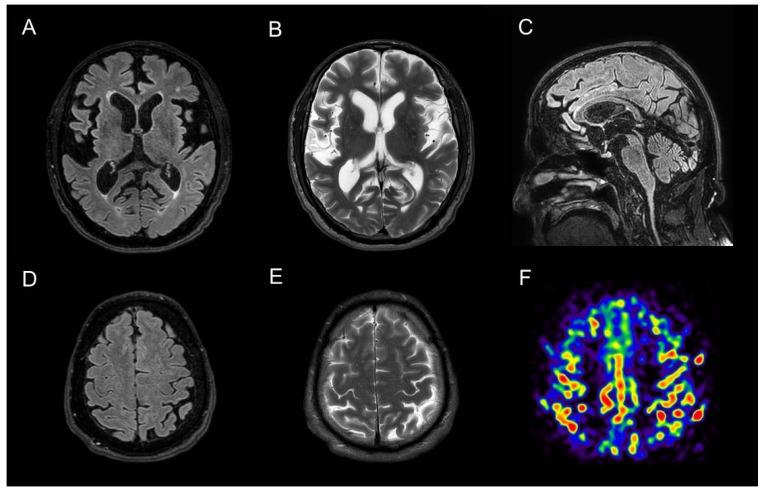
**MRI findings in a case of anti-IgLON5 disease:** (**A**,**B**) axial T1-weighted and T2-weighted sequences demonstrate marked temporal atrophy with relative preservation of other cortical regions; (**C**) sagittal T1-weighted brain MRI sequences show midbrain atrophy (the “hummingbird sign”); (**D**,**E**) axial T1-weighted and F T2-weighted sequences showed “high-convexity tight sulci”; “high-convexity tight sulci” is defined as the compression of sulci at the vertex, enlarged CSF spaces in the Sylvian fissure, and ventriculomegaly; (**F**) arterial spin labelling (ASL)-MRI documented hyperperfusion in the high-convexity area with relative preservation of other brain regions.

**Figure 2:**
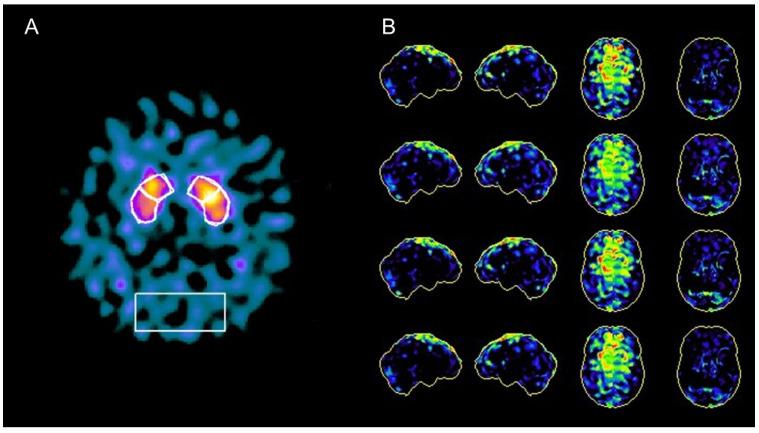
**Nuclear imaging findings in a case of anti-IgLON5 disease**: (**A**) [123I] FP-CIT SPECT showed dopaminergic nigrostriatal denervation, more prominent in the right putamen; (**B**) brain perfusion SPECT imaging documented relative hyperperfusion in the high-convexity area with relative preservation of other brain regions.

## Data Availability

Data is contained within the article.

## Supplementary Materials

The following supporting information can be downloaded at: https://www.mdpi.com/article/10.3390/diagnostics12040849/s1, Figure S1: Detection of IgLON5 antibodies using an indirect immunofluorescence cell-based assay (Euroimmun, Lubeck, Germany); patient serum (**a**) and CSF (**b**) immunoreactivity on HEK293 IgLON5 transfected cells; no serum immunoreactivity on HEK 293 nontransfected cells (**c**).
