# Supplementary material for: Neuroimaging Findings in a Patient with Anti-IgLON5 Disease: Cerebrospinal Fluid Dynamics Abnormalities
Source: Diagnostics (Basel). 2022 Mar 30;12(4):849. doi: 10.3390/diagnostics12040849 (PMC9028205; doi:10.3390/diagnostics12040849)
Supplement: Supplementary file 1 [file diagnostics-12-00849-s001.zip › diagnostics-1634152-supplementary.pdf]

diagnostics

MDPI

Interesting Images

Neuroimaging Findings in a Patient with Anti-IgLON5 Disease: Cerebrospinal Fluid Dynamics Abnormalities

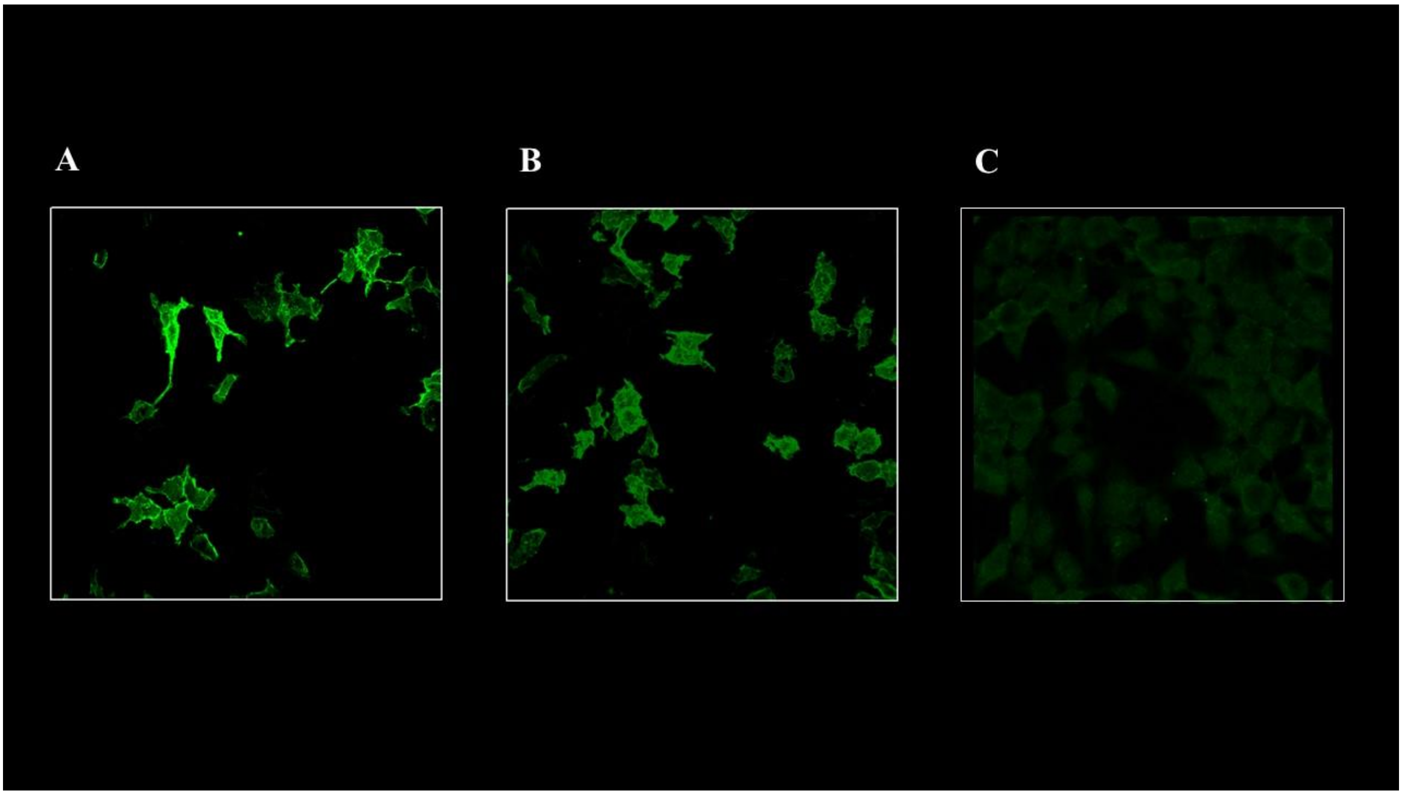

Figure S1. Detection of IgLON5 antibodies using an indirect immunofluorescence cell-based assay (Euro-immun, Lubeck, Germany); patient serum (**a**) and CSF (**b**) immunoreactivity on HEK293 IgLON5 transfected cells; no serum immunoreactivity on HEK 293 nontransfected cells (**c**).
